# Puumala Virus Infection in Family, Switzerland

**DOI:** 10.3201/eid2702.203770

**Published:** 2021-02

**Authors:** Pauline Vetter, Arnaud G. L’Huillier, Maria F. Montalbano, Fiona Pigny, Isabella Eckerle, Giulia Torriani, Sylvia Rothenberger, Florian Laubscher, Samuel Cordey, Laurent Kaiser, Manuel Schibler

**Affiliations:** University of Geneva, Geneva, Switzerland (P. Vetter, A.G. L’Huillier, F. Pigny, G. Torriani, F. Laubscher, S. Cordey, L. Kaiser, M. Schibler);; Geneva University Hospitals, Geneva (P. Vetter, M.F. Montalbano, F. Pigny, I. Eckerle, G. Torriani, F. Laubscher, S. Cordey, L. Kaiser, M. Schibler);; Geneva Centre for Emerging Viral Diseases, Geneva (P. Vetter, I. Eckerle, G. Torriani, L. Kaiser);; Spiez Laboratory, Spiez, Switzerland (S. Rothenberger);; University of Lausanne, Lausanne, Switzerland (S. Rothenberger)

**Keywords:** hantaviruses, Puumala virus, multiple organ failure, neutralization test, Hantavirus pulmonary syndrome, viruses, zoonoses, viral zoonoses, Switzerland, Russia

## Abstract

We report 3 cases of Puumala virus infection in a family in Switzerland in January 2019. Clinical manifestations of the infection ranged from mild influenza-like illness to fatal disease. This cluster illustrates the wide range of clinical manifestations of Old World hantavirus infections and the challenge of diagnosing travel-related hemorrhagic fevers.

Puumala orthohantavirus (PUUV), a species of the genus *Orthohantavirus* within the *Hantaviridae* family, is an enveloped single-strand negative-sense RNA virus (*1*). The case-fatality ratio of Old World hantaviruses ranges from 1%–10% for Dobrava-Belgrade and Hantaan orthohantaviruses to <1% for PUUV. Infection is transmitted by direct inhalation of virion-containing aerosols from rodent urine and feces. PUUV causes nephropathia epidemica, a limited form of hemorrhagic fever with renal syndrome (*1*). In Russia, 6,000–8,000 cases of hemorrhagic fever with renal syndrome are reported annually. Most cases occur in Western Russia and are caused by PUUV and Dobrava-Belgrade orthohantaviruses (*2*).

Asthenia, fever, chills, diffuse myalgia, and lumbar pain developed in a man 45 years of age 4 days after he returned to Switzerland from Samara, his hometown in central Russia (Appendix). Four days later, he sought treatment at the Geneva University Hospitals (Geneva, Switzerland) for septic shock with disseminated intravascular coagulation and kidney and liver failure. He had severe thrombocytopenia and elevated levels of C-reactive protein, procalcitonin, and leukocytes (Appendix Table 2). We transferred him to the intensive care unit for mechanical ventilation and hemodynamic support because of severe metabolic acidosis and confusion. We began treatment with broad-spectrum antimicrobial drugs, including doxycycline for possible leptospirosis. The day after admission, the patient tested positive for PUUV by real-time reverse transcription PCR (*3*) with a cycle threshold of 28. His serum sample tested positive for IgM and IgG against hantaviruses (Appendix Table 1). Shortly after his diagnosis, we administered 2 doses of 30 mg subcutaneous icatibant 6 hours apart. The patient died of multiple organ failure <60 hours after admission.

The next day, fever, lymphopenia, moderate thrombocytopenia, and hepatitis developed in the index patient’s daughter, who was 12 years of age (Appendix). She was hospitalized and tested positive for PUUV by PCR with a cycle threshold of 26. We prescribed a 5-day course of oral ribavirin starting with an initial dose of 30 mg/kg followed by 15 mg/kg every 6 hours (*4*). The viral load in plasma rapidly decreased. We did not detect viral RNA in urine (Appendix Table 3). Interstitial nephropathy briefly developed and subsided; she was discharged without sequelae after 7 days.

The wife of the index patient had had influenza-like symptoms in Russia during the week before her husband’s illness. Her serum sample tested positive for IgM and IgG against hantaviruses. We used a pseudovirus-based neutralization assay to confirm serologic results (Appendix Figure 1).

We sequenced the viral genome from blood samples taken from the father (GenBank accession no. MT822196) and the daughter (GenBank accession no. MT822195) using high-throughput sequencing (Appendix Figure 2). Both sequences showed a 100% segment match and were related to PUUV sequences in GenBank from Samara (Figure), confirming that the patients were exposed there. Regular outbreaks occur in Samara (*5*), where annual rodent control measures were delayed in 2019. In Switzerland, local acquisition of PUUV is rare (*6*).

**Figure:**
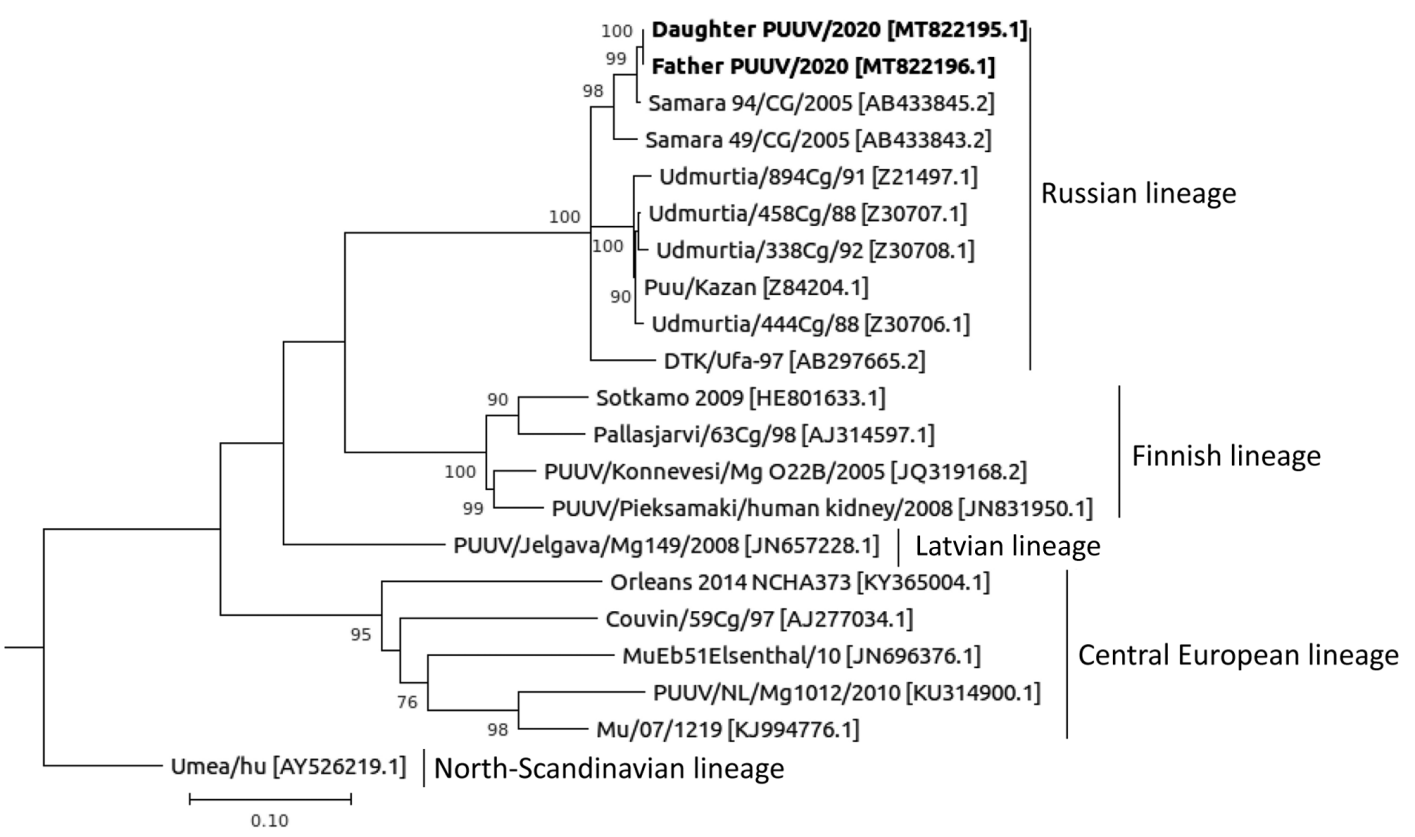
Phylogenetic tree of Puumala virus using S segment nucleotide sequences. Bold text indicates sequences isolated from family in Switzerland. GenBank accession numbers are provided in brackets. Lineages are indicated at right. Scale bar indicates number of substitutions per site.

This familial cluster highlights the wide spectrum of clinical manifestations of PUUV, which can range from an influenza-like illness (mother) to the classical nephropathy (daughter) to a rapidly fatal hemorrhagic fever with shock and multiple organ failure (father). Such a large spectrum of disease might be caused by the viral inoculum or host factors. Uncontrolled immune response and subsequent cytokine storm have been identified as key factors in the development of critical disease (*7*). Smoking, enzymatic polymorphisms, and gene variants such as HLA-B8 DRB1*03:02 (*8*) might be risk factors for severe disease, whereas HLA-B57 might have a protective effect (*9*). High procalcitonin levels, severe thrombocytopenia, increased interleukin 6 levels, and leukocytosis are known markers for severe disease.

Although specific antimicrobial drugs have been tested against PUUV infections, treatment is limited to supportive care. A small trial in Russia showed no effect of ribavirin on PUUV viral load or risk for death (*10*). We decided to treat the daughter with ribavirin because of her early diagnosis and treatment, the potential genetic factors that might predispose her to severe disease, and the emotional context of her father’s death. We treated the father with icatibant, a selective antagonist of the bradykinin type 2 receptor that reduces capillary leakage. This treatment has been used with apparent success in 2 patients with severe PUUV infection (Appendix).

PUUV usually causes limited renal disease but has a broad spectrum of clinical manifestations. Human hantavirus infections are rare in Switzerland and mostly acquired outside of the country. Physicians should consider viral hemorrhagic fevers when a patient has worsening influenza-like illness, thrombocytopenia, renal and hepatic impairment, and a plausible epidemiologic link to a region to which these viruses are endemic.

Appendix Additional information on Puumala virus infection in family, Switzerland.

## References

[1] Manigold T, Vial P. Human hantavirus infections: epidemiology, clinical features, pathogenesis and immunology. Swiss Med Wkly. 2014;144:w13937. 10.4414/smw.2014.13937 24652684

[2] Tkachenko EA, Ishmukhametov AA, Dzagurova TK, Bernshtein AD, Morozov VG, Siniugina AA, et al. Hemorrhagic fever with renal syndrome, Russia. Emerg Infect Dis. 2019;25:2325–8. 10.3201/eid2512.181649 31742540 PMC6874259

[3] Kramski M, Meisel H, Klempa B, Krüger DH, Pauli G, Nitsche A. Detection and typing of human pathogenic hantaviruses by real-time reverse transcription-PCR and pyrosequencing. Clin Chem. 2007;53:1899–905. 10.1373/clinchem.2007.093245 17717126

[4] World Health Organization. Clinical management of patients with haemorragic fever. 2016 [cited 2020 Sep 30]. https://apps.who.int/iris/bitstream/handle/10665/205570/9789241549608_eng.pdf

[5] Alexeyev OA, Suzdaltsev AA, Verkhovtsev VN, Efratova ES, Roschupkin VI. A major outbreak of hemorrhagic fever with renal syndrome in the Samara region, European Russia. Infection. 1998;26:322. 10.1007/BF02962264 9795799

[6] Fontana-Binard L, Schultze D, Rojanavisut BS, Krüger DH, Dollenmaier G, Zanetti G, et al. [First case of nephropathia epidemica acquired in Switzerland] [in French]. Rev Med Suisse. 2008;4:1572–5. 18672548

[7] Garanina E, Martynova E, Davidyuk Y, Kabwe E, Ivanov K, Titova A, et al. Cytokine storm combined with humoral immune response defect in fatal hemorrhagic fever with renal syndrome case, Tatarstan, Russia. Viruses. 2019;11:601. 10.3390/v11070601 31269734 PMC6669480

[8] Mäkelä S, Mustonen J, Ala-Houhala I, Hurme M, Partanen J, Vapalahti O, et al. Human leukocyte antigen-B8-DR3 is a more important risk factor for severe Puumala hantavirus infection than the tumor necrosis factor-alpha(-308) G/A polymorphism. J Infect Dis. 2002;186:843–6. 10.1086/342413 12198621

[9] Mustonen J, Partanen J, Kanerva M, Pietilä K, Vapalahti O, Pasternack A, et al. Association of HLA B27 with benign clinical course of nephropathia epidemica caused by Puumala hantavirus. Scand J Immunol. 1998;47:277–9. 10.1046/j.1365-3083.1998.00302.x 9519867

[10] Malinin OV, Platonov AE. Insufficient efficacy and safety of intravenous ribavirin in treatment of haemorrhagic fever with renal syndrome caused by Puumala virus. Infect Dis (Lond). 2017;49:514–20. 10.1080/23744235.2017.1293841 28276794

