# Supplementary material for: Puumala Virus Infection in Family, Switzerland
Source: Emerg Infect Dis. 2021 Feb;27(2):658–60. doi: 10.3201/eid2702.203770 (PMC7853570; doi:10.3201/eid2702.203770)
Supplement: Appendix — Additional information on Puumala virus infection in family, Switzerland. [file 20-3770-Techapp-s1.pdf]

Article DOI: https://doi.org/10.3201/eid2702.203770

Puumala Virus Infection in Family, Switzerland

Appendix

Supplementary Case Description

The index patient was not taking any medication, was a nonsmoker, and had no known relevant medical history, except for untreated limited cutaneous psoriasis. Two days before admission, he sought medical attention because of asthenia, and was diagnosed with hypertension and discharged without treatment. A few hours later, he developed an influenza-like illness characterized by fever, chills, diffuse myalgia, and lumbar pain. The next day, during a second visit, blood tests showed hemoconcentration and severe thrombocytopenia (50 G/L). Blood cultures were collected and intravenous ceftriaxone was started for a suspected urinary tract infection because of proteins and hemoglobin at urinary dipstick, despite the absence of leukocytes and nitrites. The patient was admitted 24 hours later, 4 days after symptom onset, to Geneva University Hospitals' emergency room presenting with signs of septic shock, with disseminated intravascular coagulation marked by profound thrombocytopenia in addition to kidney and liver failure.

Both C-reactive protein and procalcitonin were elevated and white blood cell count increased progressively (Appendix Table 1). Because of severe metabolic acidosis and confusion, the patient was immediately transferred to the intensive care unit for orotracheal intubation, mechanical ventilation, and hemodynamic support. Broad-spectrum antibiotic therapy, including meropenem, vancomycin, and clindamycin was initiated because septic shock of bacterial origin was suspected. The patient's wife reported a rodent invasion around Samara, their hometown in Russia, so doxycycline was added to treat potential leptospirosis.

Supplementary Case Investigations

The bacterial analysis of urine and blood cultures had negative results. A screening for multidrug resistant bacteria in stools and skin swabs also returned negative results. Samples

tested negative for the Asian hantavirus panel and Crimean–Congo hemorrhagic fever virus by RealStar CCHF RT-PCR Kit 1.0 (Altona, https://www.altona-diagnostics.com).

A full body computed tomography (CT) scan showed bi-basal pulmonary consolidations and diffuse ground-glass opacities associated with "crazy-paving" infiltrates and bilateral pleural effusions. No abdominal lesions were observed. Parieto-occipital leptomeningeal enhancements were observed on the cerebral CT scan.

A transthoracic echocardiography showed hyperdynamic cardiac function with a left ventricular ejection fraction estimated between 70% and 75%. No sign of valvulopathy and no pericardial effusion were observed.

Methods

Sequencing and Phylogenetic Tree Construction

Viral genome sequences were recovered from the father's (GenBank accession no. MT822196) and the daughter's (GenBank accession no. MT822195) blood by high-throughput sequencing on a HiSeq 4000 platform (Illumina, https://www.illumina.com) by using an RNA protocol previously published (*2*). To construct the phylogenetic tree (Figure 1), the maximum likelihood method and the Tamura 3-parameter model (*3*) were used and analyses were conducted in MEGA X (*4*). The tree is rooted using the "JN657228.1" sequence (Latvian genetic lineage).

Immunofluorescence Assay

We used Hantavirus Mosaic 1, (Euroimmun, https://www.euroimmun.com) according to the manufacturer's instructions to perform immunofluorescence assays (Appendix Table 1). Samples from the mother were taken approximately 3 weeks after the onset of symptoms.

Puumala Virus Neutralization Assay

Serum samples were inactivated by incubation for 30 min at 56°C and serial 2-fold dilutions were incubated with vesicular stomatitis virus with Puumala virus glycoprotein pseudotype virus (*5*). Residual infectivity was determined by counting green fluorescent protein–positive VeroE6 cells and expressed as a percentage of infected cells (Appendix Figure 1).

References

1. Vaheri A, Strandin T, Jääskeläinen AJ, Vapalahti O, Jarva H, Lokki ML, et al. Pathophysiology of a severe case of Puumala hantavirus infection successfully treated with bradykinin receptor antagonist icatibant. Antiviral Res. 2014;111:23–5. PubMed https://doi.org/10.1016/j.antiviral.2014.08.007

2. Petty TJ, Cordey S, Padioleau I, Docquier M, Turin L, Preynat-Seauve O, et al. Comprehensive human virus screening using high-throughput sequencing with a user-friendly representation of bioinformatics analysis: a pilot study. J Clin Microbiol. 2014;52:3351–61. PubMed https://doi.org/10.1128/JCM.01389-14

3. Tamura K. Estimation of the number of nucleotide substitutions when there are strong transition-transversion and G+C-content biases. Mol Biol Evol. 1992;9:678–87. PubMed

4. Kumar S, Stecher G, Li M, Knyaz C, Tamura K. MEGA X: molecular evolutionary genetics analysis across computing platforms. Mol Biol Evol. 2018;35:1547–9. PubMed https://doi.org/10.1093/molbev/msy096

5. Berger Rentsch M, Zimmer G. A vesicular stomatitis virus replicon-based bioassay for the rapid and sensitive determination of multi-species type I interferon. PLoS One. 2011;6:e25858. PubMed https://doi.org/10.1371/journal.pone.0025858

Appendix Table 1. Immunofluorescence assay results of family members infected with Puumala virus, Switzerland

Patient	Immunoglobulin	Dilution
Father	IgG	1:100
	IgM	1:100
Mother	IgG	1:100
	IgM	1:100
Daughter	IgG	1:100
	IgM	1:100

Appendix Table 2. Laboratory values of father with Puumala virus infection, Switzerland*

		Time after symptom onset, d			
Parameter	Reference range	4	5	6	7
Hemoglobin, g/L	140–180	**194**	**181**	133	**83**
Hematocrit, %	40.0–52.0	**53.9**	50.2	**38.5**	**25.3**
Leukocytes, 10^9 cells/L	4.0–11.0	8.6	**21.8**	**50.2**	**21.8**
% Segmented neutrophils (absolute no., 10^9 cells/L)	33.0–75.0 (1.50–7.50)	56.0/4.82	**78.5/17.11**	29.5/**14.81**	-
% Lymphocytes (absolute no., 10^9 cells/L)	15.0–60.0 (1.00–4.50)	**6.0/0.52**	**8.0**/1.74	18.5/9.29	
Platelets, 10^9 cells/L	150–350	**16**	**11**	**43**	**28**
INR		25	1.77	6.38	8.4
PTT, s	26.0–37.0	51	**65.1**	**159.8**	**>160**
Fibrinogen, g/L	1.5–3.5	2.9	1.8	**0.3**	**<0.4**
Activity Factor V, %	>70	91	**65**	**13**	**<10**
C-reactive protein, mg/L	0.00–10.00	**154**	**85**	**46**	**34**
Sodium, mmol/L	136–144	**125**	**131**	**145**	**130**
Potassium, mmol/L	3.6–4.6	4.4	4.6	4.6	**7.8**
BUN, mmol/L	3.2–7.5	4.6	**11.7**	**8.6**	5.2
Creatinine, µmol/L	62–106	108	**258**	**266**	**251**
CK total, U/L	47–222		**836**	**2'875**	**44061**
ASAT, U/L	14–50	**242**	**936**	**37'736**	N/A
ALAT, U/L	12–50	**111**	**339**	**8'811**	**6064**
Alkaline phosphatase, U/L	25–102	48	37	**121**	**235**
GGT, U/L	9–40	**115**	**47**	**46**	**103**
Total bilirubin, µmol/L	7–25	17	23	**57**	**61**
Conjugated bilirubin, µmol/L	0.5–9.5	<22	**13.9**	**33.9**	**26**
Lipase, U/L	13–60	**103**	**134**	**197**	**>600**
Lactate, mmol/L	<2	**8.4**	**5.3**	**23**	**19**
Procalcitonin, µg/L	<0.25	-	**9.5**	**1.7**	-

*Bold text indicates values outside the reference range. On day 7, no complete blood count was conducted. ALAT, alanine aminotransferase; ASAT, aspartate aminotransferase; BUN, blood urea nitrogen; CK, creatinine kinase; GGT, gamma glutamine transpeptidase; INR, international normalized ratio; PTT, partial thromboplastin time.

Appendix Table 3. Laboratory values of daughter with Puumala virus infection, Switzerland*

		Time after symptom onset, d			
Parameter	Reference range	1	3	5	7
Hemoglobin, g/L	115–155	143	140	142	130
Hematocrit, %	35.0–45.0	41.7	40.6	41.2	36.1
Leukocytes, 10^9 cells/L	4.5–13.5	**3.2**	5.6	5.2	**4.4**
% Segmented neutrophils (absolute no., 10^9 cells/L)	31.0–67.0 (1.50–7.50)	68.0/2.18	55.0/3.08	36.0/1.87	-
% Lymphocytes (absolute no., 10^9 cells/L)	22.0–51.0 (1.50–6.50)	**16.0/0.51**	23.0/**1.29**	41.0/2.13	50/2.21
Platelets, 10^9 cells/L	168–392	**100**	**67**	**113**	239
INR		1.28	1.15	1.05	1.04
PTT, s	26.0–37.0	**40.4**	**38.5**	32.6	30.4
Fibrinogen, g/L	1.5–3.5	2.9	2.9	**4.1**	3.4
C-reactive protein, mg/L	0.00–10.00	6.00			
Sodium, mmol/L	133–143	137	138	**144**	139
Potassium, mmol/L	3.5–5.1	4.3	3.8	3.7	3.7
Creatinine, µmol/L	25–52	50	57	**82**	66
ASAT, U/L	0–33	**73**	**46**	25	**50**
ALAT, U/L	0–19	28	22	23	**50**
Alkaline phosphatase, U/L	129–417	248	186	154	150
GGT, U/L	4–16	13	**26**	**29**	**27**
Total bilirubin, µmol/L	0–8	3	6	**10**	**10**
Procalcitonin, µg/L	<0.25	**0.61**	**0.68**	**0.33**	-
Urine					
Albumin, mg/L	0–10	**15**	**1002**	**367**	**11**
Creatinine, mmol/L		7.3	2.1	1.9	5.6
Protein, g/L		0.17	1.55	0.7	0.05

*Bold text indicates values outside the reference range. On day 7, no complete blood count was conducted. ALAT, alanine aminotransferase; ASAT, aspartate aminotransferase; BUN, blood urea nitrogen; CK, creatinine kinase; GGT, gamma glutamine transpeptidase; INR, international normalized ratio; PTT, partial thromboplastin time.

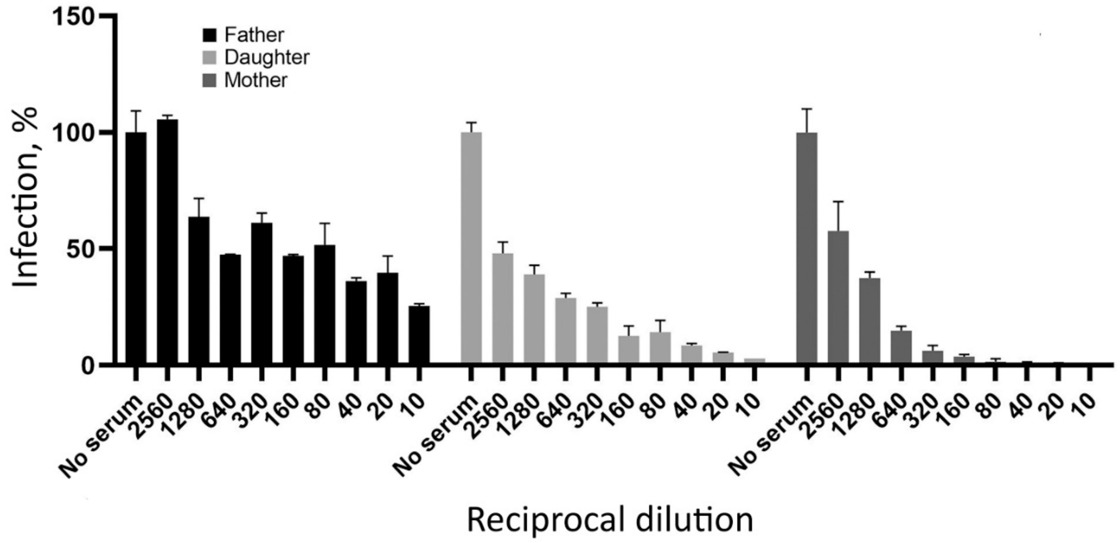

Appendix Figure 1. Neutralizing antibody levels in serum samples from members of a family with Puumala virus infection, Switzerland. Error bars indicate 95% CIs.

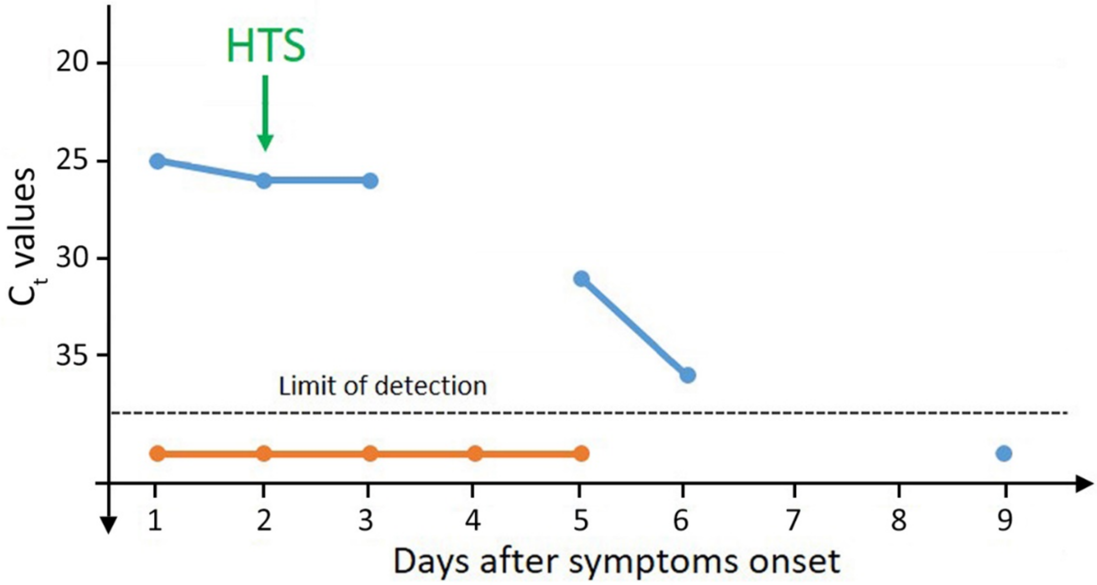

Appendix Figure 2. Cycle threshold (C_t) values of PCR of whole blood and urine samples of a girl with Puumala virus infection, Switzerland. Blue indicates whole blood samples. Orange indicates urine samples. Green arrow indicates the day of sample collection for high-throughput sequencing (HTS).
